# Chemosensitizer Effects of Coencapsulation of Curcumin and Cabazitaxel in Nanostructured Lipid Carriers in Glioblastoma Cells

**DOI:** 10.1021/acsomega.5c08442

**Published:** 2026-03-11

**Authors:** Franciely Rufino de Almeida Lima, Hélder A. Santos, Priscyla D. Marcato

**Affiliations:** 1 GNanoBio, School of Pharmaceutical Sciences of Ribeirão Preto, University of São Paulo, Ribeirão Preto, São Paulo 14040-903, Brazil; 2 Department of Biomaterials and Biomedical Technology, The Personalized Medicinee Research Institute (PRECISION), University Medical Center Groningen, University of Groningen, Ant. Deusinglaan 1, 9713 AV Groningen, The Netherlands

## Abstract

Gliomas are the most common primary tumors of the central nervous system and are associated with poor prognosis. Current therapy with Temozolomide shows limited efficacy, highlighting the need for new treatment options. Cabazitaxel (CBZ), a chemotherapeutic agent approved for prostate cancer, has shown efficacy in preclinical glioblastoma models. However, there is a need for strategies to reduce toxicity and enhance delivery. Curcumin (CUR), known for its antitumor, anti-inflammatory, and antioxidant properties, has been investigated as a potential chemosensitizer. Here, we developed and characterized a nanostructured lipid carrier (NLC) for codelivery of CBZ and CUR, evaluating its cytotoxic effect on U87MG GBM cells. The NLCs were optimized using a Box–Behnken design and exhibited a size below 150 nm, low polydispersity, and stability. The NLC–CUR/CBZ showed spherical morphology and low crystallinity that contributed to high encapsulation efficiency of both compounds (>98%). Furthermore, this system exhibited high cellular internalization and cytotoxic activity, reducing the IC_50_ by 2.418 times compared to free CBZ, and induced predominantly apoptotic cell death. The results highlight the potential of CUR and CBZ coencapsulation in NLCs for GBM treatment, providing a basis for future clinical investigations.

## Graphical abstract

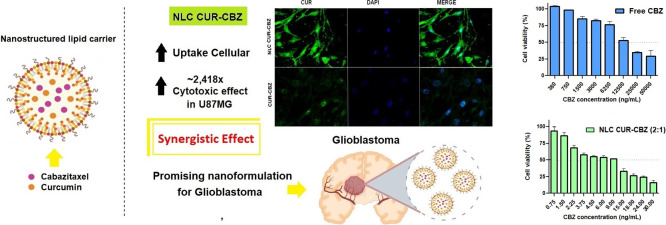

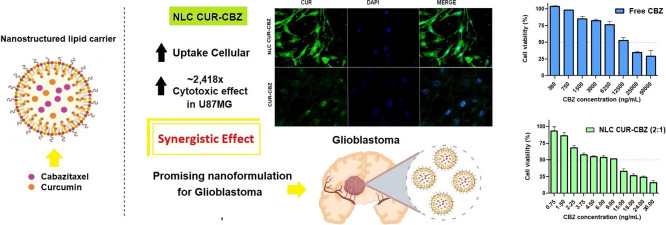

## 1 Introduction

Gliomas are the most common primary tumors of the central nervous system (CNS) and have a significant impact on patient mortality.^1^ Glioblastoma (GBM), the most prevalent and aggressive subtype in adults, accounts for approximately 54% of gliomas, with a global incidence of 3–4 cases per 100,000 people and an average survival of only 15 months.^2^ The five-year relative survival rate is less than 5%.^3^ One of the main challenges in GBM therapy is the presence of intrinsic resistance mechanisms in tumor cells, which limit the effectiveness of many chemotherapeutics. Consequently, there is an urgent need to develop strategies that enhance tumor cell sensitivity to treatment.^4,5^

Cabazitaxel (CBZ), a taxane clinically approved for prostate cancer, has emerged as a promising alternative for GBM due to its high lipophilicity and potent antitumoral activity. However, systemic administration of CBZ is often associated with neurotoxicity and other adverse effects, which limit its clinical application. To overcome these drawbacks, nanostructured drug delivery systems have been developed to enhance therapeutic performance while minimizing side effects.^4^ The encapsulation of drugs such as CBZ in nanocarriers can protect the drug from degradation, reduce toxicity, prolong circulation time, and promote accumulation in tumor tissue.^5–7^

Combining conventional chemotherapeutic drugs with natural bioactive compounds has gained increasing attention, as this approach can potentiate antitumor efficacy and overcome drug resistance. Natural compounds can sensitize cells, providing synergistic anticancer effects that may improve treatment efficacy and allow dose reduction of chemotherapeutics, minimizing potential side effects.^8–14^

Curcumin (CUR), a polyphenolic compound from *Curcuma longa*, exhibits antitumor, antioxidant, and anti-inflammatory properties, and can sensitize tumor cells to chemotherapeutic drugs, enhancing their efficacy.^8,9,13,15–18^

Studies have demonstrated synergistic effects between CUR and different chemotherapeutics, including temozolomide (TMZ), paclitaxel, and docetaxel, leading to enhanced apoptosis and inhibition of tumor growth.^19,20^ For instance, Chen et al. (2022) reported that CUR/CBZ-loaded lipid–polymer hybrid nanoparticles significantly increased cytotoxicity in prostate cancer cells compared to the free drugs. For instance, Yogan et al. (2022) reported that CUR/CBZ-loaded lipid–polymer hybrid nanoparticles, prepared using a 2:5 CUR-to-CBZ ratio, significantly increased cytotoxicity in PC-3 prostate cancer cells compared to the free drugs.^22^ Additionally, this approach also represents a drug repositioning strategy, reducing the high costs and time associated with the development of a new molecule.^21^

Despite their therapeutic potential, both CUR and CBZ exhibit low aqueous solubility and poor bioavailability, which limit their clinical performance.^23,24^ Nanostructured lipid carriers (NLCs) represent an advantageous platform for coencapsulation of lipophilic drugs, offering high drug-loading capacity, sustained release, and long-term stability. Their physicochemical characteristics, such as particle size, surface charge, and lipid composition, play a crucial role in biological performance and therapeutic response.^7,25–27^ Furthermore, the potential of this nanoparticle to deliver compounds to the brain has been demonstrated.^28–30^ Therefore, this study aimed to develop and characterize a nanostructured lipid carrier (NLC) for the codelivery of curcumin and cabazitaxel, optimizing its physicochemical properties and evaluating its cytotoxic effects on U87MG glioblastoma cells.

## 2 Results and Discussion

### 2.1 NLC Production

Initially, a preliminary lipid miscibility study was conducted to evaluate the interaction between Crodamol SS and Miglyol 180. A complete interaction, without excess oil, was verified at a 70:30 Crodamol SS/Miglyol ratio, ensuring ideal homogeneity for the NLC formulation. Subsequently, the NLC was optimized using Box–Behnken design (BBD), a three-level experimental design tool. This approach has been used to evaluate the impact of different factors on the nanoparticle physicochemical properties while minimizing the number of experiments, conserving raw materials, and saving time.^31^

Fifteen distinct formulations were produced, including three repetitions of the central point determined by the BBD. The size of NLC ranged from 37.42 to 181.5 nm, and the polydispersity index (PdI) ranged from 0.154 to 0.446 (Table S1, Supporting Information). The Pareto charts (Figure 1), generated by the BBD, indicate that the lipid phase (solid/liquid lipid), surfactant mixture, and combination of these variables significantly influenced the nanoparticle diameter. The was significantly affected by the lipid mixture and surfactant mixture variables.

**Figure 1:**
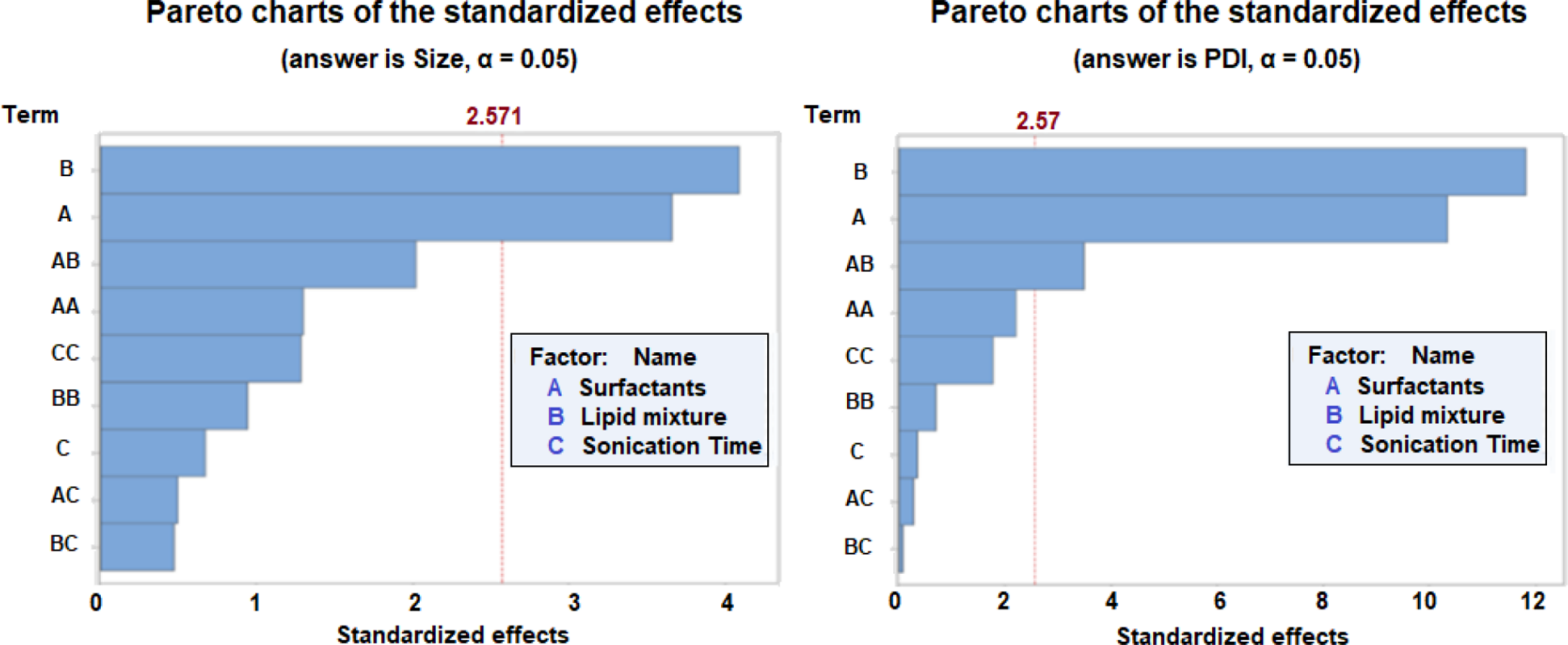
Pareto charts of the main effects of independent variables on dependent variables (percentage of lipid phase, percentage of surfactant mixture, and sonication time) on the response size and polydispersity index (PdI). The vertical line indicates the statistical significance (95% confidence level).

The equation below illustrates the impact of independent factors on the response and their interactions. Equations 1 and 2 were used to generate the 3D response surface graphs, as shown in Figure 2.

$$\mathrm{Size}=20.2-92.0\chi 1+112.6\chi 2+11.46\chi 3+39.7\chi 1^{*}\chi 1-6.49\chi 2^{*}\chi 2-0.886\chi 3^{*}\chi 3-44.1\chi 1^{*}\chi 2+0.80\chi 1^{*}\chi 3-0.17\chi 2^{*}\chi 3$$ 1

$$\mathrm{PdI}=(0.138+0.693\chi 1-0.070\chi 2-0.0435\chi 3-0.141\chi 1^{*}\chi 1+0.00276\chi 2^{*}\chi 2+0.00284\chi 3^{*}\chi 3-0.1500\chi 1^{*}\chi 2-0.0117\chi 1^{*}\chi 3+0.0058\chi 2^{*}\chi 3$$ 2

where χ1 = surfactant mixture, χ2 = lipid phase (solid/liquid lipid), χ3 = sonication time.

**Figure 2:**
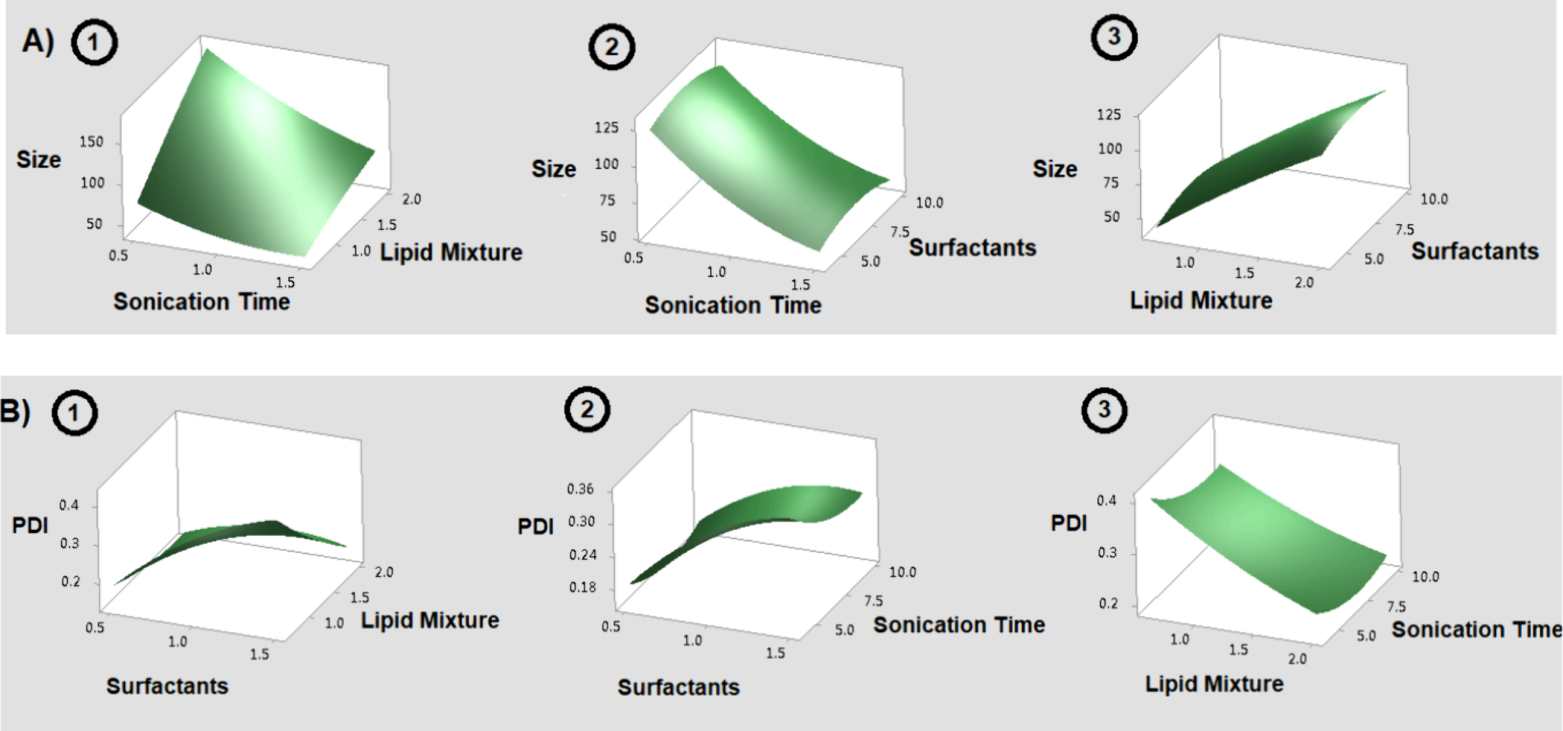
(A) Response surface plot of size showing the interaction between: (1) sonication time and lipid mixture; (2) sonication time and surfactants; and (3) surfactants and lipid mixture. (B) Response surface plot of PDI showing the interaction between: (1) surfactants and lipid mixture; (2) sonication time and surfactants and (3) sonication time and lipid mixture.

In Figure 2, it can be observed that an increase in the lipid phase results in larger particle sizes. This occurs because an increased lipid content raises viscosity, potentially leading to the formation of larger particles.^32,33^ Additionally, the percentage of surfactant mixture influenced both the size and PDI. Amphiphilic molecules reduce interfacial tension, leading to a reduction in nanoparticle diameter.^34^ Furthermore, polymeric surfactants, such as d-α-tocopheryl polyethylene glycol 1000 succinate (TPGS) and poloxamer (Pluronic), stabilize the particles through steric hindrance, contributing to preventing particle coalescence and aggregation, thereby increasing the stability of the nanoparticles.^35,36^ The sonication time did not significantly influence either dependent variable.

### 2.2 Optimization of NLC

Based on the equations provided by the BBD, we optimized the NLC by determining that the amount of each component needed to prepare a desirable NLC was 1.4% lipid phase (70:30 ratio of Crodamol SS/Miglyol), 0.67% of surfactant, and 9 min of sonication. A particle size of 120 nm and a minimum PdI were chosen because lipid nanoparticles with a diameter smaller than 200 nm are recognized to present biological advantages, including enhanced passive targeting of nanoparticles for the tumor region through the enhanced permeability and retention (EPR) mechanism, allowing nanosystems to concentrate in solid tumors.^25,26^

The minimum PdI was selected to achieve a more homogeneous formulation. The optimized NLC presented a diameter of 123.3 ± 0.5 nm, a monomodal particle size distribution profile, and a low PdI (0.195 ± 0.028; Figure 3). The optimized NLC exhibited narrow intensity-, volume-, and number-weighted DLS profiles, indicating its low polydispersity (Figure S1, Supporting Information). These values are aligned with the values predicted by the model, confirming the reliability of the model.^37,38^ The PdI is an important parameter for determining the degree of homogeneity regarding the particle size. Values less than 0.3 indicate low polydispersity of particle size.^39,40^ Thus, the optimized NLC presents low polydispersity, making it suitable for use in pharmaceutical formulations. The zeta potential reflects the surface charge of the nanoparticle. The obtained values were negative around −24 ± 1.38 mV. Since the optimized NLC has a zeta potential value different from zero and was prepared using polymeric surfactants, it can be concluded that this NLC exhibits electrosteric stability, which will contribute to the stability of the nanocarrier.^41^

**Figure 3:**
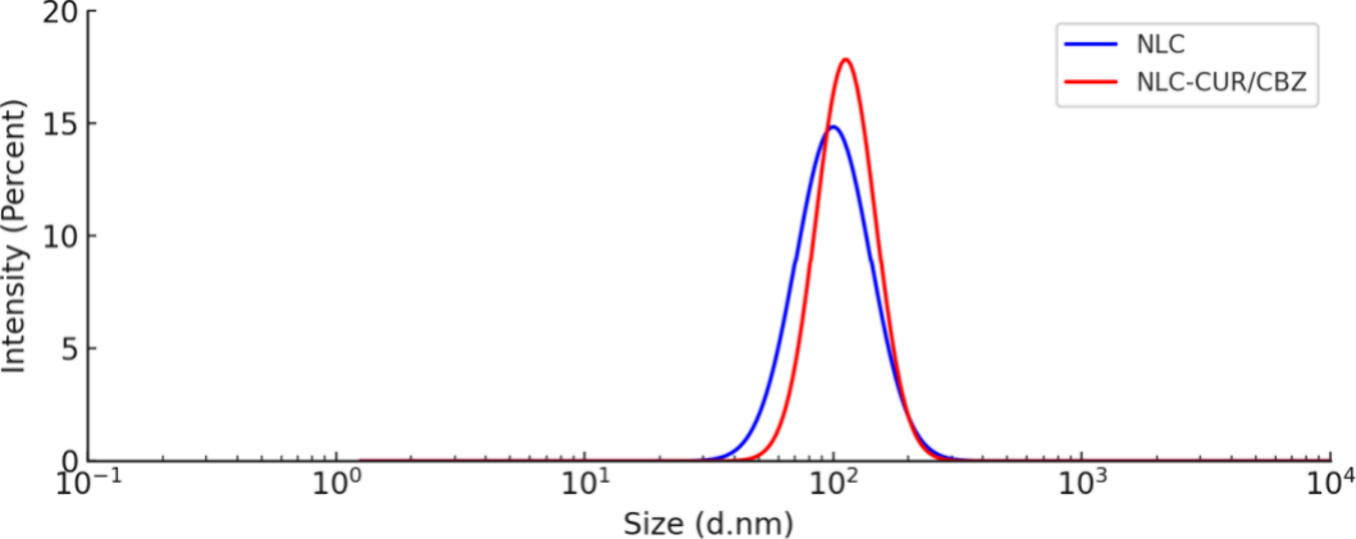
Size distribution profile of the optimized NLC (blue curve) and NLC–CUR/CBZ (red curve).

The encapsulation of CUR/CBZ (1:2) increased the particle diameter to 146.73 ± 0.23 nm, indicating the encapsulation of the compounds in the NLC. However, the formulation maintained a diameter below 200 nm, which may favor the EPR effect as discussed above.^25,27,26^ Furthermore, the NLC–CUR/CBZ exhibited a monomodal and more narrow particle size distribution profile than NLC, with lower PdI (0.155 ± 0.048) and negative zeta potential (−24 mV). The encapsulation efficiency of CUR and CBZ measured by UPLC-MS was 98.34 ± 0.8% for CUR and 99.0 ± 0.7% (*n* = 3) for CBZ. This high encapsulation efficiency may be related to the lipophilic nature of CUR and CBZ, combined with their affinity and solubility in the selected oil of the NLC. NLCs represent a second generation of lipid nanocarriers that incorporate a liquid lipid into the solid matrix; this "imperfect" structure increases drug-loading capacity and prevents the unwanted expulsion of active compounds during storage.^65^ Consequently, this combination may favor the encapsulation of CUR and CBZ in the NLC developed.^14,25^

The stability of the optimized NLC and NLC–CUR/CBZ was evaluated over 320 days (Figure 4), with no observed significant variations (*p* > 0.05) in diameter and PdI. The same stability profile was observed for the NLC–CUR/CBZ. This high stability may be attributed to the electrosteric stability of the NLC, provided by the polymeric surfactants and the negative zeta potential (−24 mV).^42,43^

**Figure 4:**
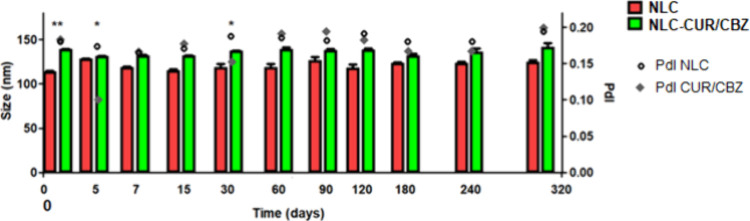
Stability profile of: NLC and NLC–CUR/CBZ over 320 days. Statistical analysis was determined by simple analysis of variance (one-way ANOVA) and Tukey’s test: (*p* > 0.05).

The results of optimization and long-term stability confirm the robustness of the developed system, representing important advantages for potential translational applications. However, it is important to recognize that the emulsification–ultrasonication method, although highly efficient at the laboratory scale, may present challenges during process scale-up. However, this scale-up can be achieved by adapting the production process to high-pressure homogenization.

### 2.3 Nanoparticle Tracking Analysis (NTA)

NTA is an important technique for nanoparticle characterization. Unlike DLS, NTA detects nanoparticles individually, distinguishing smaller particles from larger ones more effectively. Additionally, it enables the determination of nanoparticle concentration, which is crucial for both *in vitro* and *in vivo* assays.^44–46,27^

The sizes of NLC (98.6 ± 1.3 nm) and NLC–CUR/CBZ (139.4 ± 2.5 nm) did not show any statistical differences compared to the values obtained by DLS for the formulations (*p* > 0.05). Additionally, the formulation exhibited low polydispersity, as also verified by DLS, exhibiting a Span index of less than 0.6 and a narrow particle size distribution. The particle concentrations were 1.02 × 10^13^ for NLC and 1.19 × 10^13^ for NLC CUR/CBZ.

### 2.4 Transmission Electron Microscopy (TEM)

Figure 5 shows the spherical morphology of NLC and NLC–CUR/CBZ and with the presence of aggregates that can be due the dryer process.^47^

**Figure 5:**
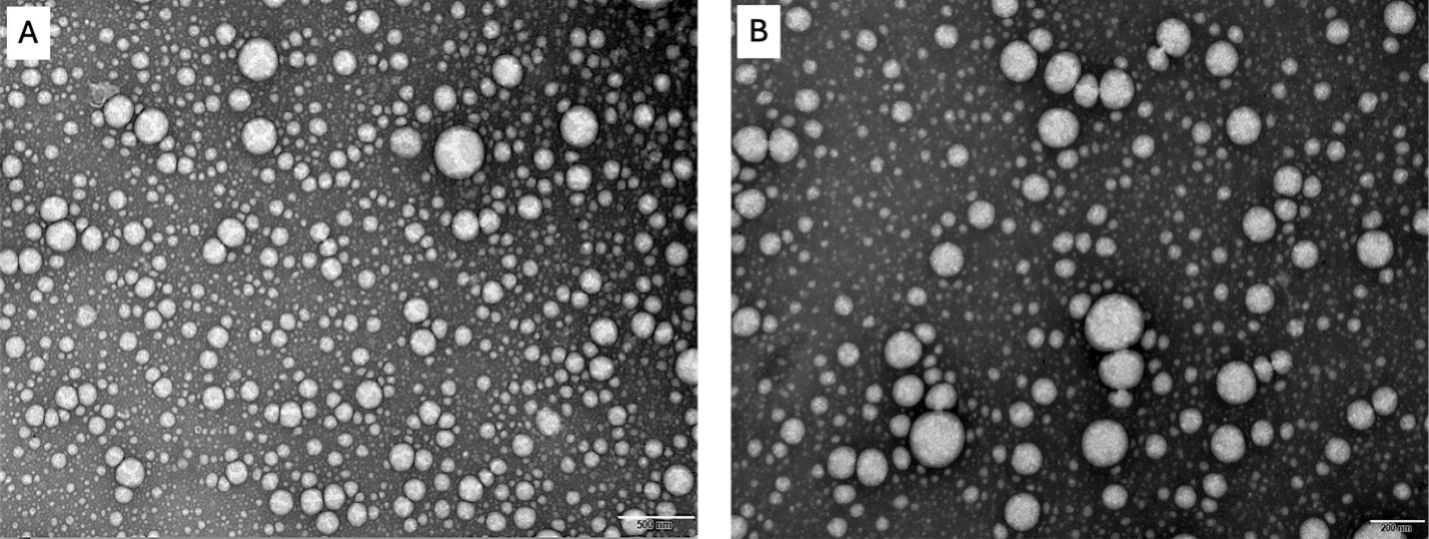
TEM images of (A) NLC (scale 500 nm); (B) NLC–CUR/CBZ (scale 200 nm).

#### 2.2.4 Differential Scanning Calorimetry (DSC) Analysis

Figure 6 shows the thermograms of the nanoparticles and raw materials. In the thermograms, it is possible to identify the melting peak of the solid lipid between 48 and 50 °C. CBZ and CUR exhibited melting peaks at 159 and 174.03 °C, respectively, both consistent with the literature.^22,24,25^ However, the absence of the melting temperature peak of CBZ or CUR in NLC–CUR/CBZ suggests that CUR and CBZ are molecularly dispersed in NLCs. Furthermore, the recrystallization index (RI%) of the NLC–CUR/CBZ was 35.97, indicating lower crystallinity of the structure and consequently better stability and higher encapsulation efficiency.^37,48,49^

**Figure 6:**
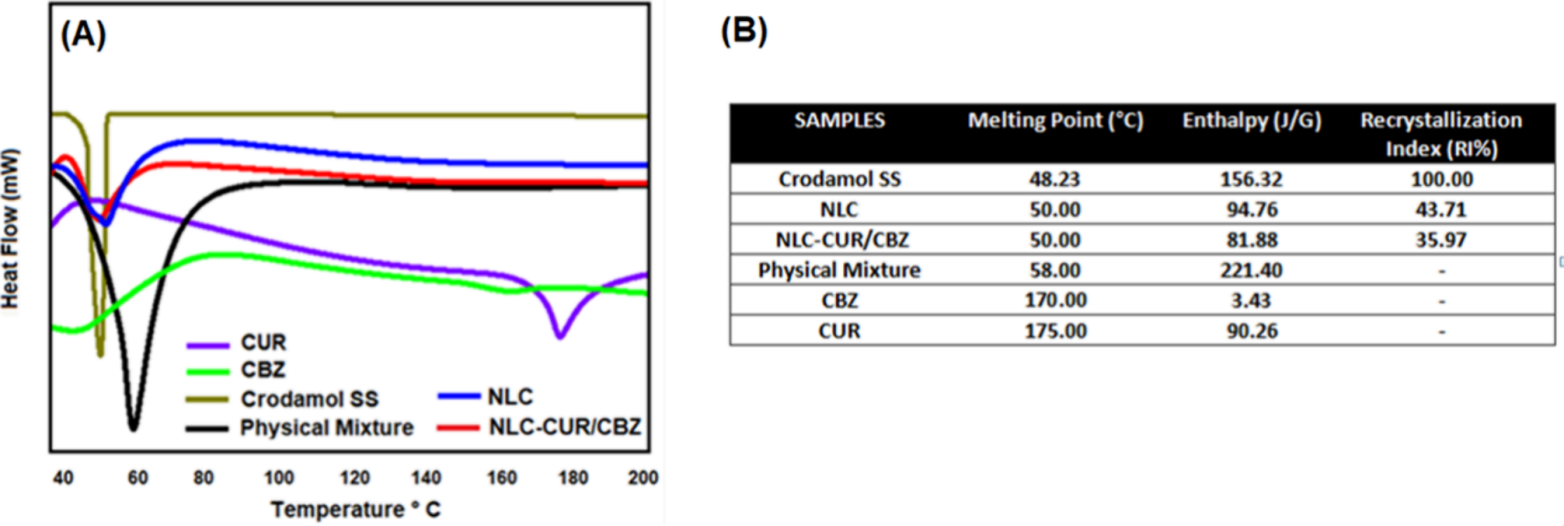
(A) Thermogram of solid lipid Crodamol SS (olive line), CBZ (green line), CUR (purple line), physical mixture (black line), NLC (blue line), and NLC–CUR/CBZ (red line). (B) Values of melting point, enthalpy, and recrystallization index obtained in DSC analysis.

### 2.5 *In Vitro* Cytotoxicity Evaluation

Evaluating a formulation in an *in vitro* model is crucial for understanding its behavior and efficacy before advancing to *in vivo* studies or clinical trials. *In vitro* models are more cost-effective and minimize the need for animal testing in the early stages of pharmaceutical development, aligning with ethical research practices.^50,51^ Thus, we assessed the cytotoxicity of free and encapsulated CUR/CBZ in glioblastoma cells U87MG.

The free CUR, free CBZ, free CUR/CBZ (1:2), and NLC–CUR/CBZ (1:2) exhibited dose-dependent cytotoxicity (Figure 7A–D). The combination of CBZ and CUR, whether free or encapsulated in NLCs, demonstrated greater cytotoxicity against U87MG cells compared to either free CBZ (IC_50_ = 15,720 ng/mL) or CUR (IC_50_ = 3280 ng/mL) alone. Qian et al. also verified the effect of curcumin in reducing the viability of U87 glioblastoma cells *in vitro* in a dose and time-dependent manner.^66^ CBZ has also been shown to exert cytotoxic effects against U87MG cells^67^ in an *in vivo* C6-induced glioblastoma rat model.^68^ The authors verified that CBZ treatment resulted in a dose-dependent reduction in tumor volume and cell number, along with modulation of oxidative stress and inflammatory markers (TNF-α and IL-10), supporting the therapeutic potential of this compound in glioblastoma treatment. Notably, the combination of CBZ/CUR in free form (IC_50_ = 13.56 ng/mL relative to CBZ) reduced the IC_50_ by approximately 1159-fold compared to free CBZ (IC_50_ = 15,720 ng/mL) (Figure 7F). Furthermore, the coencapsulation of the CUR/CBZ in NLCs (IC_50_ = 6.50 ng/mL) further enhanced the cytotoxic effect in glioblastoma cells, reducing the IC_50_ by 2418-fold compared to free CBZ (IC_50_ = 15,720 ng/mL) (Figure 7F). To assess whether this pronounced cytotoxicity could also be attributed to nanoencapsulation, CBZ was also encapsulated alone in NLCs, yielding an IC_50_ of 14.49 ng/mL (a 1084-fold reduction compared to free CBZ), which is approximately 2.26 times lower than that IC_50_ of NLC–CUR/CBZ, confirming the additional benefit of coencapsulation. This dose reduction is supported by studies on codelivery using hybrid nanoparticles. For example, Chen et al.^22^ developed aptamer-functionalized lipid–polymer hybrid nanoparticles with an average size of 121.3 ± 4.2 nm. Their study showed that codelivery of curcumin and cabazitaxel at a defined 2:5 ratio resulted in effective tumor inhibition at lower drug concentrations, enhancing tumor accumulation while reducing systemic toxicity in *in vivo* xenograft models.

**Figure 7:**
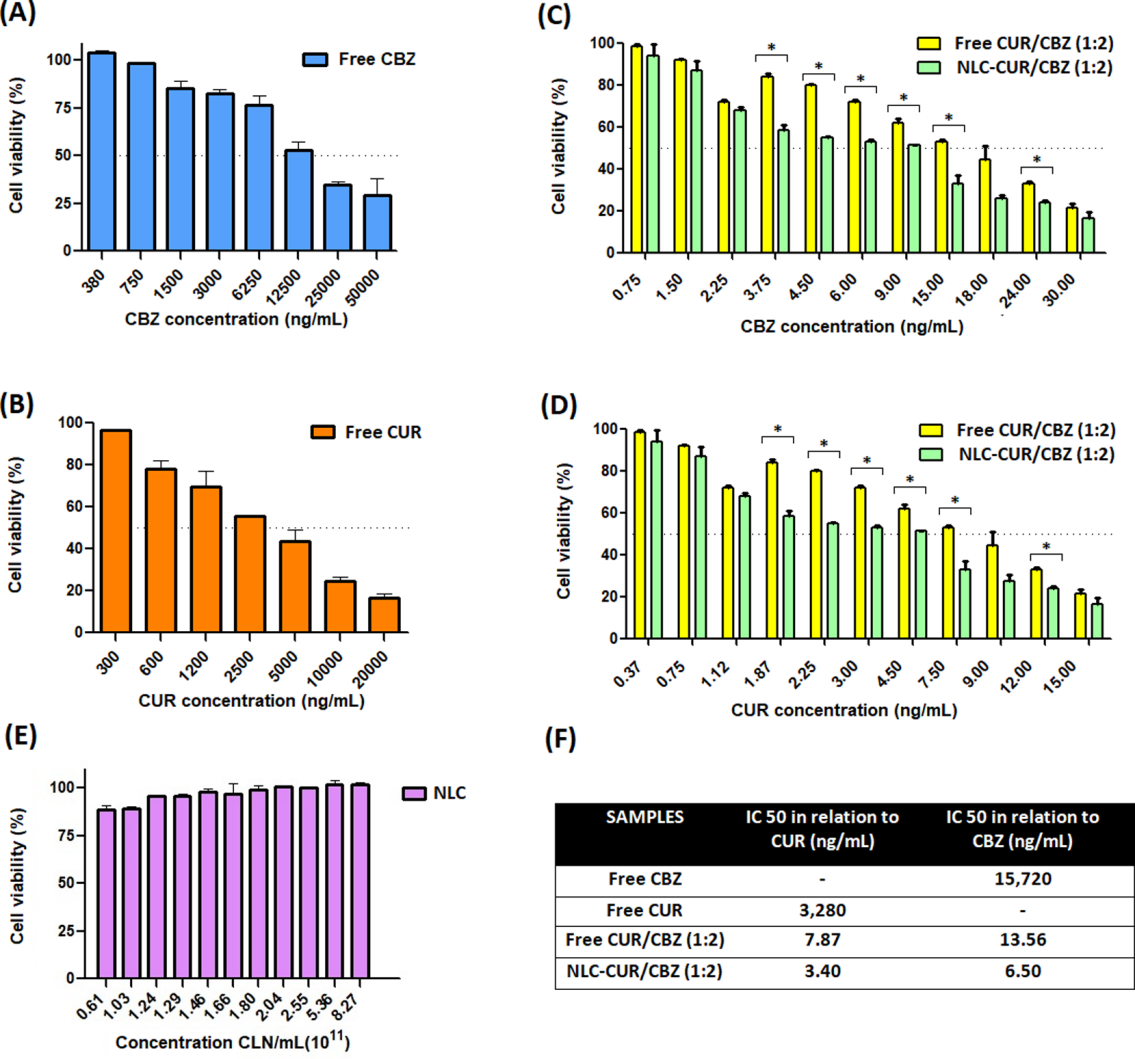
Evaluation of cytotoxicity in U87MG: (A) free CBZ; (B) free CUR; (C) free CUR/CBZ (1:2); (D) NLC–CUR/CBZ (1:2); and (E) NLC. (F) IC_50_ values of the different treatments. Statistical analysis was determined by simple analysis of variance (one-way ANOVA) and Tukey’s test:* (*p* < 0.05).

This high cytotoxicity highlights the therapeutic potential of the combination of CBZ with CUR, with the encapsulated form showing even greater cytotoxic activity. These results confirm the synergistic interaction between CBZ and CUR, aligned with previous studies that suggest a synergy effect between natural products and chemotherapeutic agents.^57^ According to Wong et al.,^69^ curcumin functions as a multitargeted agent capable of sensitizing tumor cells by modulating several key pathways, including Rb, p53, and NF-κB, which are commonly dysregulated in glioblastomas.^69,20,14^

Furthermore, coencapsulation allows the codelivery of the compound into the tumor, promoting synergistic interactions that enhance their therapeutic effects. Similar results were reported by Soni et al. (2017),^61^ where coencapsulation of chemotherapeutics in a single nanocarrier significantly improved cytotoxic potency compared to mixtures of single drug nanoparticles.^62^ Coencapsulation in NLCs also facilitates simultaneous cellular uptake, improving intracellular delivery and enhancing the cytotoxicity effect.^58,59^ The increase in biological effect observed in the coencapsulation of compounds in NLC can be enhanced mainly by improving cellular uptake of both drugs simultaneously facilitated by the nanostructure.^58,59^ The small size of NLC allows for greater interaction with cell membranes, favoring processes, such as endocytosis, where cells absorb nanocarriers more efficiently.^30,39,54^ Furthermore, the lipid structure of NLC can alter the fluidity of the cell membrane, facilitating the fusion of nanocarriers with the membrane and promoting the release of compounds.^54–58^ NLC without the compounds did not exhibit cytotoxic effects, showing a cell viability of 88.9% at the highest tested concentration (8.27 × 10^11^ NLC/mL) (Figure 7E). Temozolomide (TMZ) is the standard chemotherapeutic agent for glioblastoma, and its reported IC_50_ values in U87MG cells, according to the literature, range from 20 to 126 μg/mL (equivalent to 105–650 μM), which are substantially higher than those obtained for NLC–CUR/CBZ, thereby underscoring the therapeutic potential of the formulation developed in this study.^62–64^ Therefore, the remarkable cytotoxic enhancement observed with NLC–CUR/CBZ highlights the potential of this coencapsulated formulation as a complementary or alternative strategy to conventional TMZ therapy. However, future investigations should be carried out *in vivo* to evaluate the ability of NLC–CUR/CBZ to reach brain tumors following intravenous administration, as well as to assess their pharmacokinetics, biodistribution, and capacity to cross the blood–brain barrier.

#### 2.5.1 Evaluation of Synergism Effect of CUR/CBZ Combination

The IC_50_ values for free CUR and free CBZ were 4.28 and 15.72 ng/mL, respectively. Given the well-documented intrinsic resistance of U87MG cells to chemotherapeutic agents, the combination of anticancer drugs emerges as a rational strategy to enhance therapeutic efficacy through synergistic effects.^70^

When the 1:2 CUR/CUR ratio was tested, it was observed that in the nanoparticle formulation (IC_50_ NLC–CUR/CBZ = 6.50 ng/mL), the concentration of active compounds required to reach IC_50_ was lower than that of the combination without nanoparticles (IC_50_ free CUR/CBZ = 13.56 ng/mL), indicating a possible additive or synergistic effect. To assess this interaction, the Loewe additivity dose–effect model was applied, with the construction of an isobologram to represent synergistic, additive, and antagonistic effects.^60^ This model is based on the concept that the combination of two substances can be classified into different categories based on the Combination Index (CI), which reflects the intensity of the interaction between the compounds.^59^ The CI values were 0.116 for the CUR/CBZ 1:2 ratio with nanoparticles (1) and 0.231 for the same ratio without nanoparticles (2). According to Chou,^59^ combinations with CI < 1 are classified as synergistic, while CI > 1 indicates additive or antagonistic effects. Therefore, both 1:2 CUR/CBZ ratios (with and without nanoparticles) exhibited synergistic effects, with a more pronounced synergy observed when the compounds were associated with nanoparticles (Figure 8).^60^ The chemosensitizing effect of curcumin has been reported by Yin et al. (2014), who showed that, despite the pronounced resistance of U87MG cells to Temozolomide (TMZ), reflected by an IC_50_ of approximately 390 μg/mL, the addition of curcumin at a noncytotoxic concentration (as low as 1.25 μg/mL) was sufficient to induce synergistic interactions and significantly enhance the biological response.^71^ Complementarily, Wu et al.^72^ reported that curcumin treatment upregulated miR-146a expression in U87MG cells, which was associated with inactivation of NF-κB signaling and enhanced sensitivity to Temozolomide-induced apoptosis, supporting the role of miR-146a in curcumin-mediated chemosensitization.

**Figure 8:**
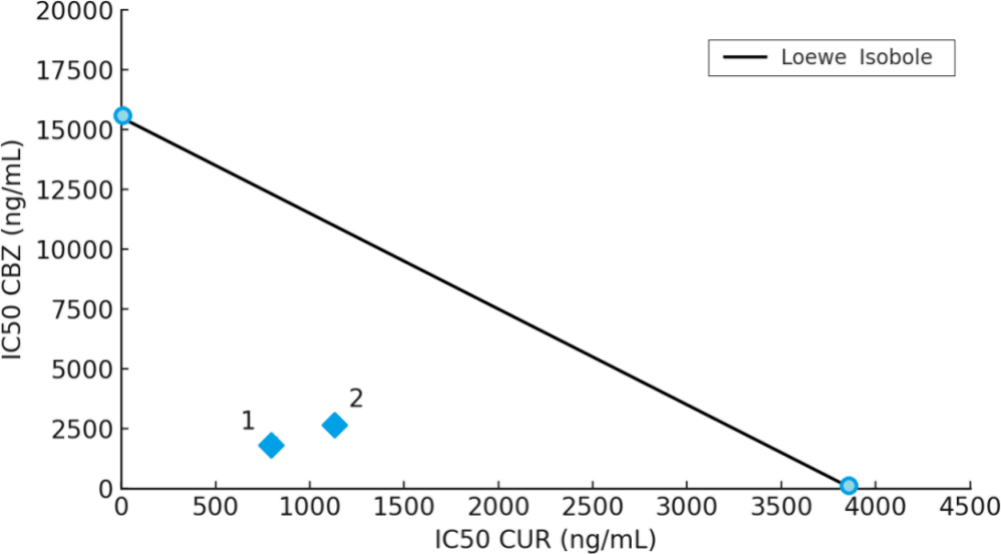
Loewe isobole for evaluating synergy and/or additivity between the CUR and CBZ. The points represent IC_50_ concentrations of CUR and CBZ in ng/mL, with (1) representing the nanoparticle formulation and (2) the non-nanoparticle formulation.

The pronounced synergistic effect may be related to the high cytotoxic activity observed for NLC–CUR/CBZ compared to free CUR/CBZ (1:2), free CBZ, or free CUR. The choice of the 1:2 CUR/CBZ ratio was based on pharmacological and formulation considerations, and it is aligned with the proportion used by Yogan et al. (2022) in prostate cancer studies (2:5, equivalent to approximately 1:2.5), which demonstrated a similarly strong synergistic interaction between the two drugs.

#### 2.5.2 Cellular Uptake Assay

The cell uptakes of NLC–CUR/CBZ and free CUR/CBZ were evaluated by confocal microscopy using CUR as a fluorescent probe (Figure 9). The green fluorescence of CUR inside the cells and the blue fluorescence of the nuclei stained with DAPI are visible after 4 h of treating U87MG cells with free CUR/CBZ and NLC–CUR/CBZ. Free CUR/CBZ showed a low green fluorescence intensity with an uneven distribution, indicating low cellular uptake of CUR. In contrast, NLC–CUR/CBZ exhibited a stronger fluorescence intensity and was efficiently internalized into the cells, as evidenced by the green fluorescence in the cells treated with nanoparticles (Figure 9). Furthermore, NLC–CUR/CBZ was effectively internalized throughout the cell, as can be seen in the merged image (Figure 9). These results highlight the ability of NLC–CUR/CBZ to penetrate glioblastoma cells and deliver compounds effectively into cancer cells, thereby enhancing the biological effect of the chemotherapeutic. Similar synergistic effects have been described in the literature for nanosystems codelivering doxorubicin (DOX) and curcumin (CUR). Kaniuk (2022) reviewed studies showing that the incorporation of both DOX and CUR in the same nanocarrier can promote synergistic interactions and improved intracellular distribution and that the intrinsic fluorescence of these compounds enables tracking of their localization and stability using confocal microscopy. These findings are consistent with the concept that nanoparticle-based codelivery systems can enhance drug bioavailability and facilitate targeted penetration of therapeutic agents into cells.^73^

**Figure 9:**
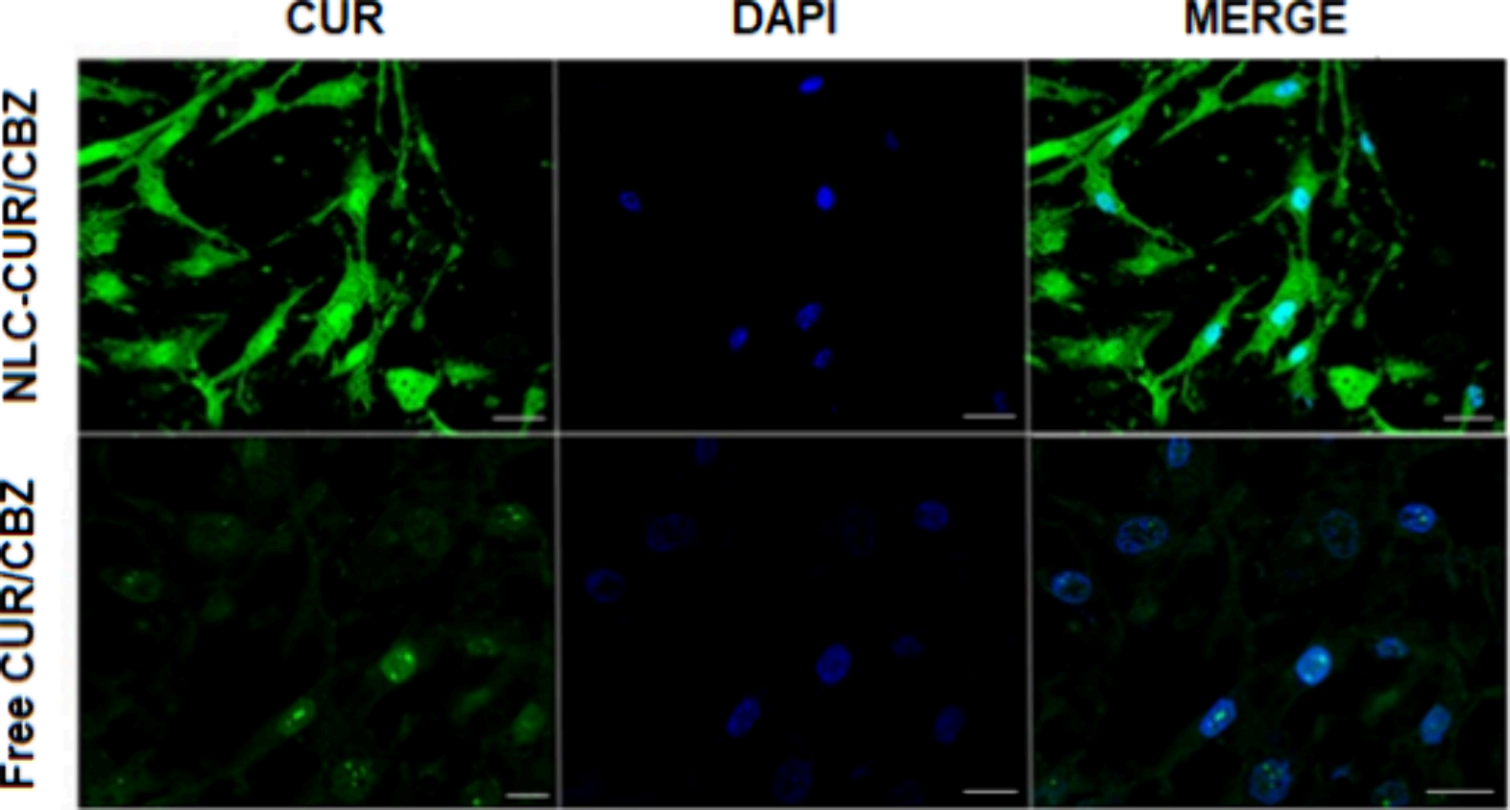
Confocal fluorescence microscopy images of U87MG cell line after 4 h treatment with NLC–CUR/CBZ (1:2) and free CUR/CBZ (1:2). Bar scale: 20 μm.

#### 2.5.3 Apoptosis and Necrosis Assay

The flow cytometry analysis demonstrated that both free CUR/CBZ and NLC–CUR/CBZ displayed a pronounced cytotoxic effect on glioblastoma cells, with NLC–CUR/CBZ exhibiting the highest cytotoxicity. These findings are consistent with the results obtained from the neutral red cytotoxicity assay. Free CUR/CBZ and NLC–CUR/CBZ significantly induced apoptosis in the treated cells compared with the negative control (Figure 10). This finding suggests that the association of CBZ with CUR possesses pro-apoptotic properties.

**Figure 10:**
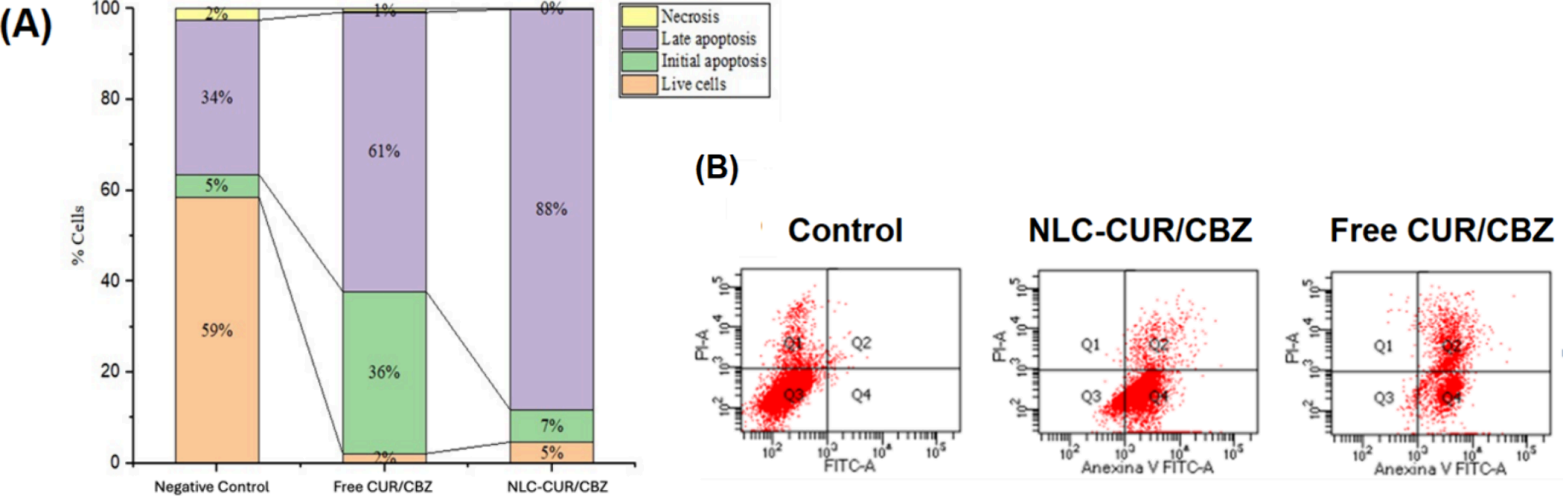
(A) D Percentage of viable, apoptotic, and necrotic U87MG cells induced by the treatments with free CUR/CBZ and NLC–CUR/CBZ. (B) Dot plot of control (U87MG cells with treatment) and those treated with NLC–CUR/CBZ or free CUR/CBZ.

CUR is known to induce apoptosis through the regulation of Bcl-2 family proteins, such as Bax and Bcl-2, in addition to activating caspases, promoting programmed cell death.^61–63^ On the one hand, it can induce oxidative stress and inhibition via NF-kB, which is crucial for cell survival.^64^ On the other hand, CBZ, a taxane, stabilizes microtubules and prevents mitosis, leading to cell death in tumor cells. It also activates apoptosis through the activation of caspases and an increase in pro-apoptotic proteins.^21,50,51^ Interestingly, NLC–CUR/CBZ was more effective in inducing late apoptosis, indicating that this formulation might be more efficient in eliminating damaged cells.^52,53^ Thus, the formulation promotes cell death predominantly via apoptosis rather than necrosis, reducing the potential inflammatory responses in the tumor microenvironment. Furthermore, free CUR/CBZ showed 1% necrotic cells, suggesting a potential risk of inflammation and other adverse reactions in the tumor microenvironment. However, no necrosis was verified in the cells treated with NLC–CUR/CBZ. Therefore, encapsulating CUR/CBZ in nanoparticles provides a more efficient delivery of active compounds, enhancing therapeutic effects while minimizing unwanted side effects. These findings have significant implications for glioblastoma treatment. Although the apoptotic and cytotoxic effects were demonstrated, further studies *in vitro*/*vitro* exploring specific molecular pathways, such as caspase activation or modulation of signaling cascades, would be valuable to deepen the understanding of CUR’s chemosensitizing role and confirm the mechanistic basis of the observed synergistic effects.

## 3 Conclusions

The developed nanostructured lipid carrier (NLC) exhibited optimized physicochemical properties, particle size below 150 nm, low polydispersity, spherical morphology, and negative zeta potential, suitable for drug delivery to the central nervous system. The formulation showed high encapsulation efficiency for both curcumin (CUR) and cabazitaxel (CBZ) (>98%), attributed to their lipophilic nature and the reduced crystallinity of the lipid matrix.

*In vitro* studies confirmed that the coencapsulation of CUR and CBZ within NLCs significantly enhanced cytotoxic and chemosensitizing effects against U87MG glioblastoma cells, demonstrating a synergistic interaction supported by the combination index (CI < 1). The simultaneous delivery of both drugs within a single nanocarrier improved cellular internalization and apoptosis induction, while minimizing necrosis.

Although apoptotic and cytotoxic effects were clearly demonstrated, future investigations should be conducted to elucidate the molecular mechanisms involved, such as caspase activation and modulation of signaling pathways. Additionally, *in vivo* studies assessing pharmacokinetics, biodistribution, blood–brain barrier penetration, and safety are essential to confirm the translational potential of this formulation.

Overall, these findings demonstrate that the NLC–CUR/CBZ system represents a promising therapeutic approach for glioblastoma treatment, combining synergistic efficacy, physicochemical stability, and strong potential for further preclinical and clinical development.

## 4 Material and Methods

### 4.1 NLC Production

#### 4.1.1 Lipid Miscibility Study

Different ratios of solid lipid (Crodamol SS, Croda) and liquid lipid (Miglyol 810, Sasol) were tested to find the optimal mix for nanostructures. This solid lipid and the oil were chosen based on the solubility of curcumin (CUR, Sigma-Aldrich) and cabazitaxel (CBZ, Health Biochem Technology Co). The solid lipid and oils were agitated at 300 rpm for 30 min at 60 °C to allow the solid lipid melt and interact with the oil. Afterward, the mixture was transferred to filter paper, and the size of oily halos was observed.

#### 4.1.2 Preparation of NLC

The NLC were prepared were prepared in one step by the emulsification-ultrasonication method. The solid lipid Crodamol SS and the oil Miglyol 810 were heated to 50–55 °C. An aqueous solution containing the surfactant Pluronic 407, lecithin, and TPGS (1:1:1) was added to the heated mixture at the same temperature as for the lipids. The hot emulsion was sonicated for 10 min (Sonics VCX 750, probe of 13 mm, 40% of amplitude), and then the dispersion was cooled at 25 °C in a water bath. The CUR (5 mg) and CBZ (10 mg) were added to the lipid phase after the solid lipid melted.

#### 4.1.3 NLC Optimization by Box–Behnken Design (BBD)

To optimize NLC, the BBD with three factors at three levels was employed. The chosen independent variables were lipid mixture, surfactant, and sonication time, while the dependent variables (response) were polydispersity index (PdI) and hydrodynamic diameter (z-average). Fifteen formulations were suggested using Minitab 18 software, including a triplicate of the central point. Table S2 (Supporting Information) presents the independent variables and their respective limits.

#### 4.1.4 CBZ and CUR Encapsulation Efficiency

The encapsulation efficiency (EE) of CUR and CBZ coencapsulated in NLC was assessed by an indirect method. The dispersion was added to the Microcon (Millipore) filter with an ultrafiltration regenerated cellulose membrane with a molecular weight cutoff of 10 000 g/mol and centrifuged at 5000 × g for 10 min. The filtrate was quantified by ultraperformance liquid chromatography (UPLC) coupled with a Single Quadrupole (SQ) mass spectrometer with an electrospray ionization (ESI) source (UPLC-MS, Waters). The system operated in negative electrospray ionization mode (ESI+) and used an Acquity UPLC BEH C18 column (50 × 2.1 mm, 1.7 μm particle size, Waters, Milford, MA, USA) at a flow rate of 0.39 mL/min and a temperature of 40 °C. The mobile phase consisted of ultrapure water with 0.1% formic acid (A) and acetonitrile (B), following a gradient elution as detailed: 60% A and 40% B (0 min), → 100% B and 0% A (3 min), → 60% A and 40% B (5 min). Each sample (10 μL, 100–800 ng/mL for both drugs) was injected under these conditions. In the mass spectrometry system, the gas flow rate was set to 650 L/h for desolvation, with a gas temperature of 400 °C. The ionization source operated at a temperature of 400 °C with a capillary voltage of 2.5 kV and a cone voltage of 60 V. These parameters ensured optimal ionization and detection of the analytes. The analytical curve of CBZ was *y* = 11,955*x* + 1192.56; *r* = 0.9956, and CUR was *y* = 166,644*x* – 1266.3; *r* = 0.9978. The EE of NLC–CUR/CBZ was determined according to eq 3 ^30^

$$\mathrm{EE}=\frac{[\mathrm{CURouCBZ}{]}_{\mathrm{inicial}}-[\mathrm{CURouCBZ}{]}_{\mathrm{filtrated}}}{[\mathrm{CURouCBZ}{]}_{\mathrm{inicial}}}\times 100$$ 3

### 4.2 Physicochemical Characterization of NLC–CUR/CBZ

#### 4.2.1 Particle size, PDI, and Zeta Potential

Particle size (z-average), PdI, and zeta potential of the NLC and NLC–CUR/CBZ were determined using a Zetasizer Nano ZS90 instrument (Malvern Instruments, United Kingdom) at a 90° angle. The samples were diluted in a 1 mM KCl solution (Sigma-Aldrich) before analysis. For stability analysis during 320 days, potassium sorbate (0.2% w/v) and sodium benzoate (0.1% w/v) were added to the formulations, and the samples were stored at (25 ± 2 °C). The particle size, polydispersity index (PdI), and zeta potential of the samples were measured on different days.

#### 4.2.2 Nanoparticle Tracking Analysis (NTA)

The NLC and NLC–CUR/CBZ size and concentration were analyzed using NanoSight NS300 equipment (Malvern, UK) equipped with a red laser (642 nm) and NTA 4.1 software. The dispersions were diluted 15,000× in ultrapure water before the analysis, and 43 to 58 particles per frame were analyzed.

#### 4.2.3 Transmission Electron Microscopy (TEM)

The morphological characterization of the NLC and NLC–CUR/CBZ was conducted using transmission electron microscopy (TEM) technique, utilizing the JEM-100 CXII microscope (JEOL, Japan), operating at 80 kV. The sample was diluted in water and stained with 2% uranyl acetate for 30 s, then placed on a carbon-coated copper grid, forming a thin liquid film that was dried for analysis.

#### 4.2.4 Differential Scanning Calorimetry (DSC)

Thermal properties of the nanoparticles and raw materials were analyzed using a Jade differential scanning calorimeter (PerkinElmer, Waltham, MA, USA). Lyophilized NLC and NLC–CUR/CBZ, physical mixture, CUR, and CBZ (4–6 mg) were added to sealed aluminum pans and heated from 20 to 200 °C under a nitrogen atmosphere at a flow rate of 10 °C/min. The recrystallization index (RI) was calculated according to eq 4 below:^31^

$$\%\mathrm{IR}=\frac{\Delta H_{\mathrm{nanoparticle}}}{\Delta H_{\mathrm{bulklipid}}+\mathrm{fraction}\;\mathrm{of}\;\mathrm{lipid}\;\mathrm{phase}}\times 100$$ 4

### 4.3 *In Vitro* Cytotoxicity Evaluation

#### 4.3.1 Cytotoxicity in Monolayer Model

The U87MG cell line (obtained from Rio de Janeiro Cell Bank, BCRJ 0241) was cultured in DMEM medium supplemented with 10% fetal bovine serum (FBS) and 1% antibiotics (penicillin and streptomycin) and maintained at 37 °C in a humidified atmosphere with 5% CO_2_. Cells were seeded in 96 well plates (1 × 10^4^ cells per well) and incubated for 24 h prior to treatment with different concentrations (0.3–20 μg/mL) of free CUR, free CBZ (0.38–50 μg/mL), NLC–CUR/CBZ, and CUR/CBZ free mixture (0.75–30 ng/mL of CBZ) for 48 h. The negative, solvent, and positive controls consisted of a 10% FBS culture, 0.5% (v/v) DMSO, and 10% (v/v) DMSO, respectively. After a 24 h incubation period, cytotoxicity in cultures was assessed using the neutral red uptake assay. Absorbance was measured at 540 nm with a microplate reader (BioTek).

#### 4.3.2 Cellular Uptake Assay

Cellular uptake of nanoparticles in U87MG cells was evaluated by using confocal laser scanning microscopy. CUR was used as a fluorescent probe in the free form and encapsulated in NLC–CUR/CBZ. U87MG cells (6 × 10^5^ cells/mL) were cultured in a 4-well plate and incubated for 72 h. After this period, cells were treated with free and encapsulated CUR/CBZ (3.4 ng/mL CUR/6.8 ng/mL CBZ) for 4 h. Subsequently, cells were washed with PBS and stained with 2 drops of Fluoroshield with DAPI (Sigma-Aldrich) for 30 min. U87MG cells were analyzed using a laser scanning confocal microscope (Leica SP8) equipped with wavelength filters for DAPI (λ = 405 nm, blue) and CUR (λ = 530 nm, green).

#### 4.3.3 Apoptosis and Necrosis Assay

U87MG cells (6.0 × 10^5^ cells/mL) were seeded in a six-well plate and incubated for 24 h. The cells were then treated with free CUR/CBZ and NLC–CUR/CBZ at concentrations of 6.50 ng/mL CUR and 13.0 ng/mL CBZ. After 24 h, cells were harvested with trypsin, centrifuged, and washed twice with cold PBS. The cell pellets were resuspended in 100 μL of buffer, stained with Annexin V and propidium iodide (PI), and analyzed by flow cytometry using the FACSCanto (BD, USA) with FACSDiva software version 6.4.1 (BD, USA). Excitation/emission wavelengths of 490/525 nm were used for Annexin V and 488/670 nm for PI. For each experimental point, a cell density of 1 × 10^4^ cells/mL was analyzed in triplicate. Total apoptosis, including both early and late apoptosis, and necrosis were quantified.

### 4.4 Evaluation of the Synergism Effect of CUR/CBZ Combination

To assess the combined effect of CBZ and CUR on antitumor activity, cytotoxicity assays were performed in U87MG cells in quadruplicate, and IC_50_ values for free CUR and CBZ were obtained as reference parameters for their combination. The combination of CUR and CBZ in a 1:2 ratio was tested both in free form and encapsulated in nanoparticles, with IC_50_ values calculated for each condition. The combination index (CI) was calculated using the Chou–Talalay method according to eq 5 using Excel software,^59^ with CI < 1 indicating synergy, CI ≈ 1 indicating additivity, and CI > 1 indicating antagonism.^60^ with CI < 1 indicating synergy, CI ≈ 1 indicating additivity, and CI > 1 indicating antagonism.^60^ An isobologram was constructed, where points below the Loewe isobole indicate synergy, and points above indicate antagonism, suggesting higher doses for the combined therapeutic effect.^66^

$$\mathrm{CI}=\frac{(D)\mathrm{Cur}}{(\mathrm{Dx})\mathrm{Cur}}+\frac{(D)\mathrm{CBZ}}{(\mathrm{Dx})\mathrm{CBZ}}=\frac{(D)\mathrm{Cur}}{\left(\mathrm{Dm})\mathrm{Cur}{[}^{\frac{\mathrm{fa}}{\left(1-\mathrm{fa}\right)}}\right]}+\frac{(D)\mathrm{CBZ}}{\left(\mathrm{Dm})\mathrm{CBZ}{[}^{\frac{\mathrm{fa}}{\left(1-\mathrm{fa}\right)}}\right]}$$ 5

### 4.5 Statistical Analysis

The results were presented as the mean ± standard deviation (SD) from at least three independent experiments. Statistical analysis was conducted using ANOVA followed by Tukey’s multiple comparisons test in GraphPad Prism version 5.0. Differences with *p*-values less than 0.05 or 0.01 were considered statistically significant.

## Supplementary Material



## Notes

Independent variables applied in the Box–Behnken experimental design and their respective limits (Table S1); complete experimental design matrix with the corresponding dependent responses (particle size and polydispersity index) (Table S2); and the dynamic light scattering (DLS) size distribution histograms of nanostructured lipid carriers, presented according to intensity-, volume-, and number-based distributions (Figure S1) (PDF)
