# Supplementary material for: Chemosensitizer Effects of Coencapsulation of Curcumin and Cabazitaxel in Nanostructured Lipid Carriers in Glioblastoma Cells
Source: ACS Omega. 2026 Mar 11;11(11):17223–34. doi: 10.1021/acsomega.5c08442 (PMC13019242; doi:10.1021/acsomega.5c08442)
Supplement: Supplementary file 1 [file ao5c08442_si_001.pdf]

Supporting information

Chemosensitizer effects of co-encapsulation of curcumin and cabazitaxel in nanostructured lipid carriers in glioblastoma cells

Franciely Rufino de Almeida Lima[1], Hélder A. Santos[2], Priscyla D. Marcato[1,*]

Table S1. Independent variables and their limits**.**

Independent variables	Limits		
	Low (-1)	Medium (0)	High (+1)
Lipid Phase (%)	0.5	1	1.5%
Surfactant Mixture (%)	0.6	1.3	2%
Sonication Time (min)	4	7	10

Table S2. Independent variables and obtained responses for dependent variables in BBD.

Run	Surfactants Mixture (%)	Lipid Phase (%)	Sonication Time (min)	Size (nm)	PDI
1	0.5	2	7	117.2	0.212
2	1	2	4	92.38	0.251
3	1	1.3	7	84.95	0.293
4	1	1.3	7	38.35	0.421
5	1.5	0.6	7	113.8	0.204
6	0.5	1.3	10	83.59	0.140
7	0.5	0.6	7	55.41	0.262
8	1.5	1.3	10	117.7	0.197
9	0.5	1.3	4	113.1	0.170
10	1	2	10	87.02	0.226
11	1	1.3	7	40.13	0.446
12	1	0.6	4	63.92	0.325
13	1.5	1.3	4	37.42	0.355

14	1	0.6	10	75.97	0.225
15	1.5	2	7	181.5	0.154

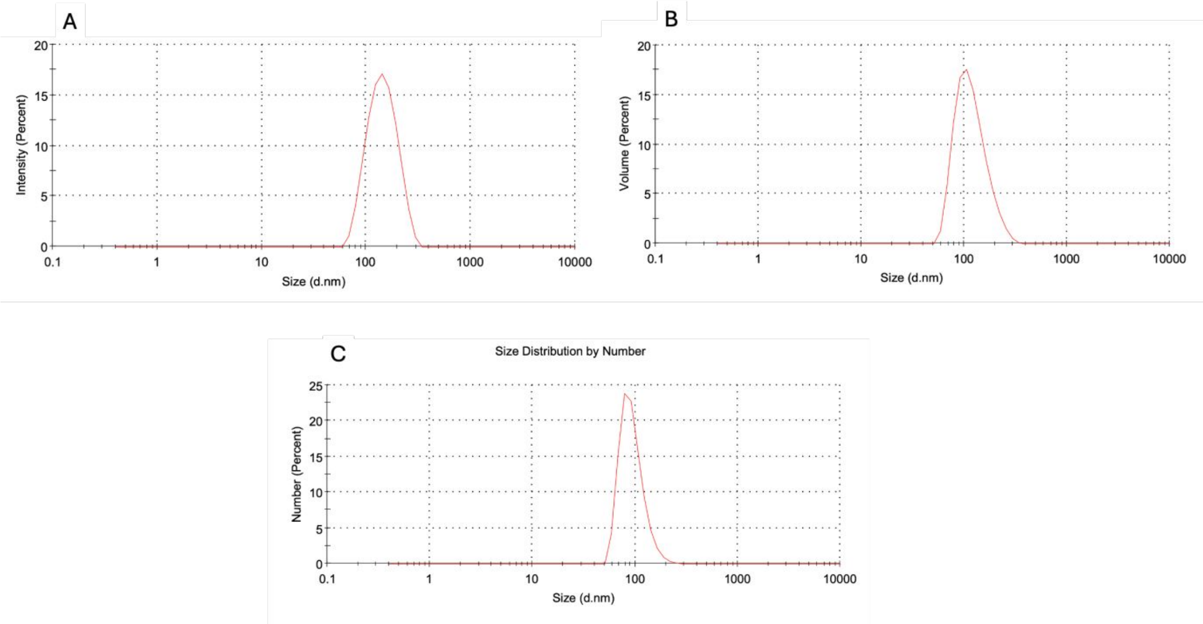

Figure S1: DLS size distribution histograms of NLC based on: (A) intensity; (B) volume; and (C) number.
